# Supplementary material for: Usefulness of Tree Species as Urban Health Indicators
Source: Plants (Basel). 2021 Dec 17;10(12):2797. doi: 10.3390/plants10122797 (PMC8709473; doi:10.3390/plants10122797)
Supplement: Supplementary file 1 [file plants-10-02797-s001.zip › plants-1466358-supplementary.pdf]

Supplementary Material Table S1. The Canonical Discriminant Analysis output of studied tree species based on the elemental concentrations of leaves.

Parameters	DF1	DF2	DF3	DF4	DF5	DF6	DF7
Eigenvalue	184.258	23.420	8.387	5.334	5.102	1.400	0.956
% of Variance	80.5	10.2	3.7	2.3	2.2	0.6	0.4
Cumulative %	80.5	90.7	94.4	96.7	99	99.6	100
Canonical Correlation	0.997	0.979	0.945	0.918	0.914	0.764	0.699
Wilks' Lambda	0.000	0.000	0.001	0.006	0.035	0.213	0.511
Chi-square	166.502	111.674	78.122	54.610	35.227	16.237	7.045
df	105	84	65	48	33	20	9
Significance	<0.001	0.023	0.127	0.238	0.363	0.702	0.632

Supplementary Material Table S2. The instrumental conditions for ICP-OES for trace elemental analysis.

Wavelengths/nm	**Al:** 396.152
	**Ba**: 455.403
	**Ca:** 422.673
	**Cd**: 226.502
	**Co**: 228.615
	**Cr**: 267.716
	**Cu**: 324.754
	**Fe**: 238.204
	**K**: 766.491
	**Li**: 670.783
	**Mg**: 279.553
	**Mn**: 257.610
	**Na**: 589.592
	**Ni**: 216.555
	**Pb**: 220.353
	**Sr**: 407.771
	**Zn**: 213.857
Background correction	fitted
Number of replicates	3
Pump speed (rpm)	15
Rinse time (sec)	30
Nebulizer flow (l/min)	0.7
Analysis pump rate (rpm)	15
Plasma mode	dual
RF power (kW)	1.2
Read time (sec)	5
Plasma flow (l/min)	12.0
Sample introduction	SPS 4 autosampler
